# Interactions of platelets with obesity in relation to lung cancer risk in the UK Biobank cohort

**DOI:** 10.1186/s12931-023-02561-9

**Published:** 2023-10-17

**Authors:** Sofia Christakoudi, Konstantinos K. Tsilidis, Evangelos Evangelou, Elio Riboli

**Affiliations:** 1 Department of Epidemiology and Biostatistics, School of Public Health, Imperial College London, St Mary’s Campus, Norfolk Place, London, W2 1PG UK; 2 Department of Inflammation Biology, School of Immunology and Microbial Sciences, King’s College London, London, UK; 3 Department of Hygiene and Epidemiology, University of Ioannina School of Medicine, Ioannina, Greece

**Keywords:** Platelet count, MPV, PDW, Obesity, Interaction, Lung cancer

## Abstract

**Background:**

Platelet count (PLT) is associated positively with lung cancer risk but has a more complex association with body mass index (BMI), positive only in women (mainly never smokers) and inverse in men (mainly ever smokers), raising the question whether platelets interact with obesity in relation to lung cancer risk. Prospective associations of platelet size (an index of platelet maturity and activity) with lung cancer risk are unclear.

**Methods:**

We examined the associations of PLT, mean platelet volume (MPV), and platelet distribution width (PDW) (each individually, per one standard deviation increase) with lung cancer risk in UK Biobank men and women using multivariable Cox proportional hazards models adjusted for BMI and covariates. We calculated Relative Excess Risk from Interaction (RERI) with obese (BMI ≥ 30 kg/m^2^), dichotomising platelet parameters at ≥ median (sex-specific), and multiplicative interactions with BMI (continuous scale). We examined heterogeneity according to smoking status (never, former, current smoker) and antiaggregant/anticoagulant use (no/yes).

**Results:**

During a mean follow-up of 10.4 years, 1620 lung cancers were ascertained in 192,355 men and 1495 lung cancers in 218,761 women. PLT was associated positively with lung cancer risk in men (hazard ratio HR = 1.14; 95% confidence interval (CI): 1.09–1.20) and women (HR = 1.09; 95%CI: 1.03–1.15) but interacted inversely with BMI only in men (RERI = − 0.53; 95%CI: − 0.80 to − 0.26 for high-PLT-obese; HR = 0.92; 95%CI = 0.88–0.96 for PLT*BMI). Only in men, MPV was associated inversely with lung cancer risk (HR = 0.95; 95%CI: 0.90–0.99) and interacted positively with BMI (RERI = 0.27; 95%CI = 0.09–0.45 for high-MPV-obese; HR = 1.08; 95%CI = 1.04–1.13 for MPV*BMI), while PDW was associated positively (HR = 1.05; 95%CI: 1.00–1.10), with no evidence for interactions. The associations with PLT were consistent by smoking status, but MPV was associated inversely only in current smokers and PDW positively only in never/former smokers. The interactions with BMI were retained for at least eight years of follow-up and were consistent by smoking status but were attenuated in antiaggregant/anticoagulant users.

**Conclusions:**

In men, PLT was associated positively and MPV inversely with lung cancer risk and these associations appeared hindered by obesity. In women, only PLT was associated positively, with little evidence for interaction with obesity.

**Supplementary Information:**

The online version contains supplementary material available at 10.1186/s12931-023-02561-9.

## Background

Platelets can promote carcinogenesis by releasing growth and angiogenic factors and extracellular vesicles, which induce changes in stromal and tumour cells [1]. The lung, as well as a major cancer cite [2], is a major site of terminal platelet production from circulating megakaryocytes [3]. Correspondingly, platelet count (PLT) is not only higher at the time or shortly prior to lung cancer diagnosis [4, 5], but lung cancer risk remains consistently higher in individuals with higher PLT for at least ten years prior to diagnosis [6].

Although general obesity, as reflected in body mass index (BMI), is associated with higher risk of venous thromboembolism [7], BMI shows an inverse, smoking-related association with lung cancer risk [8, 9]. Moreover, we have previously shown in UK Biobank that BMI is associated positively with PLT only in women, most strongly in never smokers, but inversely in men, most strongly in ever smokers [10]. This raises the question whether PLT interacts with obesity in relation to lung cancer risk.

PLT is only one aspect of platelet pathology and does not provide information about platelet functionality, while platelet size could be indicative of platelet activity, as thrombotic conditions are associated with large mean platelet volume (MPV) [11] and large platelet variability (platelet distribution width, PDW) [12]. Platelet size could also provide information about platelet maturity, as platelet precursors are larger than mature platelets [13]. Therefore, examining platelet size in conjunction with PLT could provide more information about potential mechanistic pathways than examining PLT in isolation. Little is known, however, about associations of platelet size with lung cancer risk. The available studies are few, with small number of patients, focused on lung cancer diagnosis and prognosis, and reporting mainly higher MPV and PDW in lung cancer patients compared to healthy controls [14, 15]. To our knowledge, there are no studies evaluating prospectively associations of MPV or PDW with lung cancer risk.

In this study, we used data from the UK Biobank cohort to investigate the prospective associations of PLT, MPV, and PDW with lung cancer risk and their interactions with obesity in men and women.

## Methods

### Study population

UK Biobank includes half a million participants registered with the National Health Service, which were aged 40 to 70 years at recruitment (years 2006 to 2010) and were living within 40 km of an assessment centre in England, Scotland, and Wales [16]. In this study, we included participants with self-reported white ancestry, due to limited numbers from other ethnic groups, and excluded participants with prevalent cancer at recruitment, missing or extreme anthropometric measurements, mismatch between the genetic and self-reported sex, missing platelet measurements, using antihemorrhagic agents, and pregnant women (total excluded 91,253 (18.2%), see Additional file 1: Table S1 for details).

### Lung cancer ascertainment

Cancer cases in UK Biobank are ascertained based on linkage to the national cancer registry of the United Kingdom. The outcome of interest was first primary lung cancer diagnosed after recruitment, defined with code C34 from the 10th version of the International Statistical Classification of Diseases (ICD10) and malignant behaviour (behavioural code 3 or 5), defined as in [17]. Follow-up was censored at the date of diagnosis for first primary incident lung cancers with rare morphology (codes 8710, 8800, 8801, 8990, 9050, 9120, 9133, 9591, 9680, 9699) and for cancers in locations other than the lung (excluding non-melanoma skin cancers but including skin squamous-cell carcinomas). For all participants remaining cancer-free, follow-up was censored at the earlier of the date of death or 31st March 2020 (last complete cancer registry).

### Platelet and anthropometric measurements

Blood samples were collected at recruitment in EDTA (ethylenediaminetetraacetic acid) vacutainers, throughout the day (irrespective of fasting status) and were analysed within 24 h of blood draw [18]. PLT (10^9^/L) was measured directly, while MPV (fL) and PDW (%) were derived from scatter plots and histograms of platelet size on Beckman Coulter LH750 automated analysers. We log-transformed all platelet parameters, to mitigate right-skewness of the distributions.

The anthropometric assessments were obtained at recruitment by trained technicians according to established protocols [19]. We calculated BMI as weight (kg) divided by height squared (m).

### Analytical approach

We examined as exposures platelet parameters (PLT, MPV, or PDW) on a standardised continuous scale (z-scores, value minus mean, divided by standard deviation (SD) after log-transformation), interpreting hazard ratios (HR) per one SD increase. We examined men and women separately, due to the pronounced sex differences in the associations of obesity with platelet parameters [10]. We examined platelet parameters individually, because they are correlated substantially with each other [10] and could be biologically related and hence not independent.

We explored heterogeneity in groups according to BMI (normal weight BMI = 18.5 to < 25 kg/m^2^; overweight BMI = 25 to < 30 kg/m^2^; obese BMI = 30 to < 45 kg/m^2^) and used the data augmentation method of Lunn and McNeil [20] to compare HR estimates for men vs women and for obese vs normal/overweight within each sex.

To evaluate additive interactions, we calculated the Relative Excess Risk from Interaction (RERI) [21] in fully adjusted models including a platelet-obesity cross-classification, defined by dichotomising to high/low at ≥ median (sex-specific) PLT (234.0 men; 261.4 women), MPV (9.17 men; 9.25 women), and PDW (16.50 men; 16.38 women), and dichotomising BMI ≥ 30 kg/m^2^ (obese):

$$\text{RERI}=\text{HR}_{\text{High-High}}-\text{HR}_{\text{High-Low}}-\text{HR}_{\text{Low-High}}+1$$

We obtained confidence intervals and p-values for RERI with the delta method applied in function **nlcom** in STATA-13 [22].

To evaluate multiplicative interactions between platelet parameters and BMI on a continuous scale, we used the Wald test for the corresponding interaction term, examining each platelet-BMI pair in a separate model with adjustment for covariates.

### Statistical models

We used STATA-13 for the statistical analyses and R version 4.1.3 [23] for data management. Tests of statistical significance were two-sided. Given the exploratory nature of the analysis and the higher power requirements for testing interactions, we used nominal statistical significance (p < 0.05).

We obtained HRs and 95% confidence intervals (CI) from delayed-entry Cox proportional hazards models, which account for left-truncation and are conditional on surviving cancer-free to cohort recruitment. We used age as the underlying time scale, with origin at the date of birth, entry time at the date at recruitment, and exit time at the earliest of the date of diagnosis of the first primary incident cancer, or death, or last complete follow-up. We stratified all models by age at recruitment (five-year categories), region of the assessment centre, and for women, a combined variable reflecting menopausal status and hormone replacement therapy (HRT) use (defined similarly to [24]), with four categories (pre-menopausal women; post/unknown menopause never HRT; post/unknown menopause past HRT; post/unknown menopause current HRT). We adjusted all models for BMI and height (sex-specific z-scores), weight change within the year preceding recruitment (weight loss, stable weight, weight gain), smoking status and intensity (never smoked; just tried; former occasional; former regular quit ≥ 20 years; former regular quit ≥ 10 years; former regular quit < 10 years; current occasional; current regular ≤ 10 cigarettes/day; current regular > 10 cigarettes/day), alcohol consumption (≤ 3 times/month; ≤ 4 times/week; daily), physical activity (less active; moderately active; very active), Townsend deprivation index quintiles (as proxy of socio-economic status), family history of cancer (no cancer; breast/bowel/prostate; lung cancer), time of blood collection (< 12:00; 12:00 to < 16:00; ≥ 16:00), fasting time (0–2 h; 3–4 h; ≥ 5 h), self-reported diabetes (assuming that all participants with self-reported diabetes were treated), use of lipid lowering drugs, antihypertensive drugs, antiaggregant/anticoagulants, non-steroidal anti-inflammatory drugs (NSAID), and paracetamol (defined as no/yes similarly to [25]). Non-smoking covariates were selected a priori, based on previous reports for associations with platelet parameters and lung cancer risk. The adjustment for drug use aimed to account for exogenous influences on metabolic and inflammatory conditions, thrombosis, and liver fat accumulation and function, which can affect platelet parameters and platelet activity (see further details in Additional file 1). Antiaggregant/anticoagulant users included > 90% aspirin users and < 7% anticoagulant-only users, which were added to this group as they were too few to be considered separately. Information for all covariates was obtained at recruitment (initial assessment visit). We replaced missing values for covariates (< 2%, see Additional file 1: Table S2 for details) with the median category for each sex.

To confirm correlations between platelet parameters in this study, we calculated partial Pearson correlation coefficients adjusted for all covariates, except region of the assessment centre, and using age, Townsend deprivation index, time of blood collection, and fasting time as continuous variables, and smoking with categories never, former, and current smoker.

### Sensitivity analyses

To examine the influence of adjustment on the observed associations and interactions, we used models stratified by age only and models additionally adjusted only for smoking status and intensity. To examine the potential influence of reverse causality, we excluded participants with less than two years and less than eight years of follow-up and lagged the entry date with two or eight years, correspondingly, to condition on surviving cancer free to later than recruitment.

Last, we examined the consistency of our findings in groups according to smoking status (never/former smokers combined and individually never, former, and current smokers), as smoking can affect platelet parameters [26], and according to antiaggregant/anticoagulant use, as this would alter platelet function. For exposures on a continuous scale, we compared HR estimates between groups with the augmentation method of Lunn and McNeil [20].

## Results

### Cohort characteristics

During a mean follow-up of 10.4 years, 1620 lung cancers were ascertained in 192,355 men and 1495 lung cancers were ascertained in 218,761 women (Table 1). Less than a quarter of participants were obese. Men were more likely to be current smokers and antiaggregant/anticoagulant users, while women were more likely to be never smokers. PLT and MPV were lower in men, while PDW was lower in women. MPV and PDW were correlated inversely with PLT and positively with each other, as previously reported [10].

**Table 1:** Anthropometric characteristics and platelet parameters of study participants

Characteristics	Men	Women
Cohort	192,355	218,761
Cases	1620	1495
Cases: per 100,000	842	683
Follow-up (years)^a^	10.4 (2.3)	10.6 (2.1)
Age at recruitment (years)^a^	57.2 (8.1)	56.9 (8.0)
BMI (kg/m^2^)^a^	27.8 (4.0)	26.9 (4.8)
BMI categories		
Normal weight^b^	47,997 (25.0)	88,030 (40.2)
Overweight^b^	95,905 (49.9)	81,481 (37.2)
Obese^b^	48,453 (25.2)	49,250 (22.5)
Smoking status		
Never smoker^b^	93,527 (48.6)	129,366 (59.1)
Former smoker^b^	75,404 (39.2)	70,219 (32.1)
Current smoker^b^	23,424 (12.2)	19,176 (8.8)
Antiaggregant/anticoagulant use^#^		
Yes^b^	40,138 (20.9)	23,470 (10.7)
Platelet parameters		
PLT (*10^9^/L)^c^	232 (145–370)	260 (165–409)
PDW (%)^c^	16.6 (15.6–17.6)	16.4 (15.5–17.4)
MPV (fL)^c^	9.23 (7.40–11.49)	9.31 (7.46–11.62)
Correlation of platelet parameters		
PLT-MPV^d^	− 0.47	− 0.49
PLT-PDW^d^	− 0.37	− 0.36
MPV-PDW^d^	0.40	0.41

*BMI* body mass index, *MPV* mean platelet volume, Normal weight – BMI = 18.5 to < 25 kg/m^2^, Overweight – BMI = 25 to < 30 kg/m^2^, Obese – BMI = 30 to < 45 kg/m^2^, *PDW* platelet distribution width, *PLT* platelet count

^a^Mean (standard deviation); ^b^number (percent from total per sex); ^c^geometric mean (95% reference range); ^d^Partial Pearson correlation coefficient; ^#^aspirin users were 36,558 (91.1% of antiaggregant/antiaggregant users) in men and 21,784 (92.8%) in women; participants using only anticoagulants were also included in this group, because they were too few to be considered separately: 2685 (6.7% of the group) in men and 1170 (5.0%) in women

Comparisons between sexes were performed with unpaired-samples t-test for BMI on a continuous scale and log-transformed platelet parameters and χ^2^-test for BMI categories, smoking categories, and antiaggregant/anticoagulant use. All differences were significant at p < 0.0001

Summaries of all covariates are presented in Additional file 1: Table S2

### Associations of platelet parameters with lung cancer risk

**Fig. 1:**
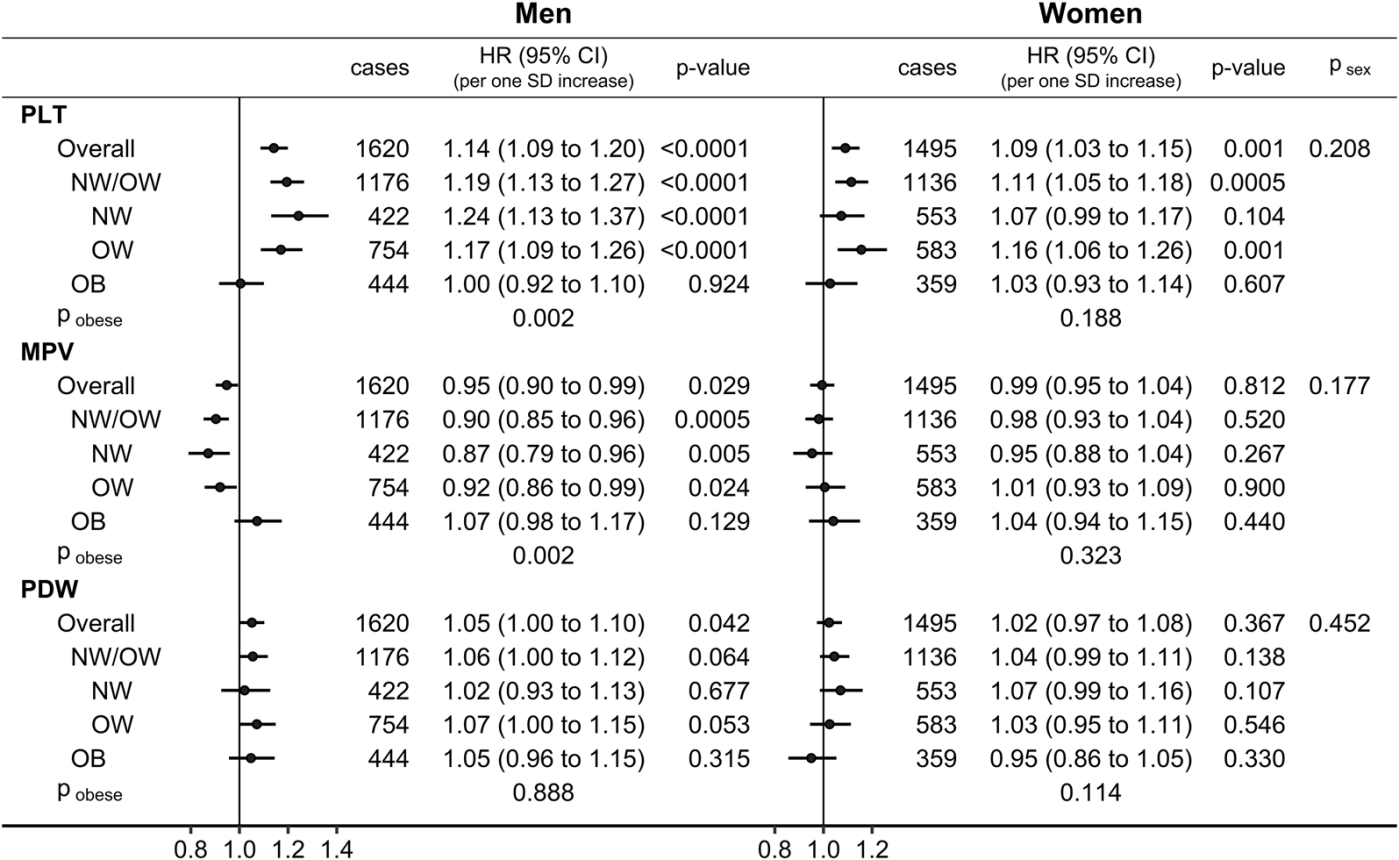
Associations of platelet parameters (continuous scale) with lung cancer risk. *BMI* body mass index, *Cases* number of lung cancer cases per group, *CI* confidence interval, *HR* hazard ratio, *MPV* mean platelet volume, *NW* normal weight BMI = 18.5 to < 25 kg/m^2^; *OW* overweight BMI = 25 to < 30 kg/m^2^; *OB* obese BMI = 30 to < 45 kg/m^2^; p-value Wald test for the individual term, *PDW* platelet distribution width, *PLT* platelet count, *SD* standard deviation. Cox proportional hazards models with exposure either PLT, MPV, or PDW (sex-specific z-scores, value minus mean divided by standard deviation after log-transformation), stratified by age at recruitment, region, and in women, menopausal status and hormone replacement therapy use, and adjusted for BMI and height (sex-specific z-scores), recent weight change, smoking status and intensity, alcohol consumption, physical activity, Townsend deprivation index, family history of cancer, time of blood collection, fasting time, diabetes, and use of lipid-lowering drugs, antihypertensive drugs, antiaggregant/anticoagulants, non-steroidal anti-inflammatory drugs, and paracetamol. p_obese_ & p_sex_ – p-value comparing the association with PLT, MPV, or PDW between OB and NW/OW or between men and women with the augmentation method of Lunn and McNeil [20]

PLT was associated positively with lung cancer risk in men (HR = 1.14; 95%CI: 1.09–1.20 per one SD increase) and women (HR = 1.09; 95%CI: 1.03–1.15), more specifically in normal weight and overweight and not in obese participants, but with clear evidence for heterogeneity between BMI categories only in men (p_obese_ = 0.002) (Fig. 1). Although there was no heterogeneity by sex with nominal statistical significance, MPV was associated inversely with lung cancer risk only in men (HR = 0.95; 95%CI: 0.90–0.99), more specifically for normal weight and overweight but not for obese men (p_obese_ = 0.002). PDW was associated weakly positively with lung cancer risk also only in men (HR = 1.05; 95%CI: 1.00–1.10).

### Interactions of platelet parameters with obesity

High-PLT was associated positively with lung cancer risk for BMI < 30 kg/m^2^ in men and women, but the risk in high-PLT-obese participants was lower than expected by an additive effect mainly for men (RERI = − 0.53; 95%CI: − 0.80 to − 0.26 for high-PLT-obese) and less for women, and there was a matching inverse multiplicative interaction only in men (HR = 0.92; 95%CI = 0.88–0.96 for PLT*BMI) (Table 2). High-MPV was associated inversely with lung cancer risk for BMI < 30 kg/m^2^ only in men, and there was consistent evidence for positive additive and multiplicative interactions of MPV with BMI only in men (RERI = 0.27; 95%CI = 0.09–0.45 for high-MPV-obese; HR = 1.08; 95%CI = 1.04–1.13 for MPV*BMI). High-PDW was associated positively with lung cancer risk for BMI < 30 kg/m^2^ also only in men, but with no evidence for additive or multiplicative interactions.

**Table 2:** Additive and multiplicative interactions of platelet parameters with obesity

Platelet	BMI		Men			Women	
Parameter	(kg/m^2^)	Cases	HR (95% CI)	p-value	Cases	HR (95% CI)	p-value
*PLT*							
PLT-Low	< 30	457	Reference		494	Reference	
PLT-High	< 30	719	1.39 (1.23 to 1.56)	< 0.0001	642	1.24 (1.10 to 1.40)	0.0004
PLT-Low	≥ 30	243	1.17 (1.00 to 1.38)	0.052	165	0.90 (0.75 to 1.08)	0.268
PLT-High	≥ 30	201	1.03 (0.87 to 1.22)	0.743	194	0.93 (0.79 to 1.11)	0.449
RERI			− 0.53 (− 0.80 to − 0.26)	0.0001		− 0.21 (− 0.45 to 0.03)	0.090
PLT*BMI		1620	0.92 (0.88 to 0.96)	0.0003	1495	0.97 (0.92 to 1.02)	0.295
*MPV*							
MPV-Low	< 30	635	Reference		562	Reference	
MPV-High	< 30	541	0.87 (0.77 to 0.98)	0.017	574	1.02 (0.91 to 1.15)	0.709
MPV-Low	≥ 30	187	0.78 (0.66 to 0.92)	0.004	158	0.77 (0.64 to 0.93)	0.006
MPV-High	≥ 30	257	0.92 (0.79 to 1.07)	0.282	201	0.87 (0.74 to 1.03)	0.110
RERI			0.27 (0.09 to 0.45)	0.004		0.08 (− 0.13 to 0.28)	0.471
MPV*BMI		1620	1.08 (1.04 to 1.13)	0.0006	1495	1.05 (1.00 to 1.11)	0.046
*PDW*							
PDW-Low	< 30	540	Reference		563	Reference	
PDW-High	< 30	636	1.13 (1.01 to 1.27)	0.038	573	1.03 (0.91 to 1.16)	0.640
PDW-Low	≥ 30	181	0.93 (0.78 to 1.10)	0.388	163	0.84 (0.70 to 1.01)	0.061
PDW-High	≥ 30	263	1.02 (0.87 to 1.19)	0.826	196	0.82 (0.69 to 0.97)	0.021
RERI			− 0.04 (− 0.26 to 0.18)	0.728		− 0.05 (− 0.26 to 0.16)	0.628
PDW*BMI		1620	1.02 (0.98 to 1.07)	0.353	1495	0.99 (0.94 to 1.04)	0.579

*BMI* body mass index, *Cases* number of lung cancer cases per group, *CI* confidence interval, *HR* hazard ratio, *MPV* mean platelet volume, *p-value* p-value from Wald test for the individual term for cross-classification categories, or p-value for RERI derived with the delta method, or p-value from Wald test for the multiplicative interaction term, *PDW* platelet distribution width, *PLT* platelet count, *RERI* relative excess risk from interaction (additive interaction)

Cox proportional hazards models including, for each platelet parameter indicated in the headings, a cross-classification with obese (RERI) or an interaction term with BMI on a continuous scale (*) (sex-specific z-scores, value minus mean divided by standard deviation, after log-transformation for platelet parameters), stratified by age at recruitment, region, and in women, menopausal status and hormone replacement therapy use, and adjusted for height, recent weight change, smoking status and intensity, alcohol consumption, physical activity, Townsend deprivation index, family history of cancer, time of blood collection, fasting time, diabetes, and use of lipid-lowering drugs, antihypertensive drugs, antiaggregant/anticoagulants, non-steroidal anti-inflammatory drugs, and paracetamol

Platelet parameters were dichotomised (high/low) with respect to ≥ median (sex-specific): PLT (234.0 men; 261.4 women), MPV (9.17 men; 9.25 women), PDW (16.50 men; 16.38 women)

### Sensitivity analyses

**Fig. 2:**
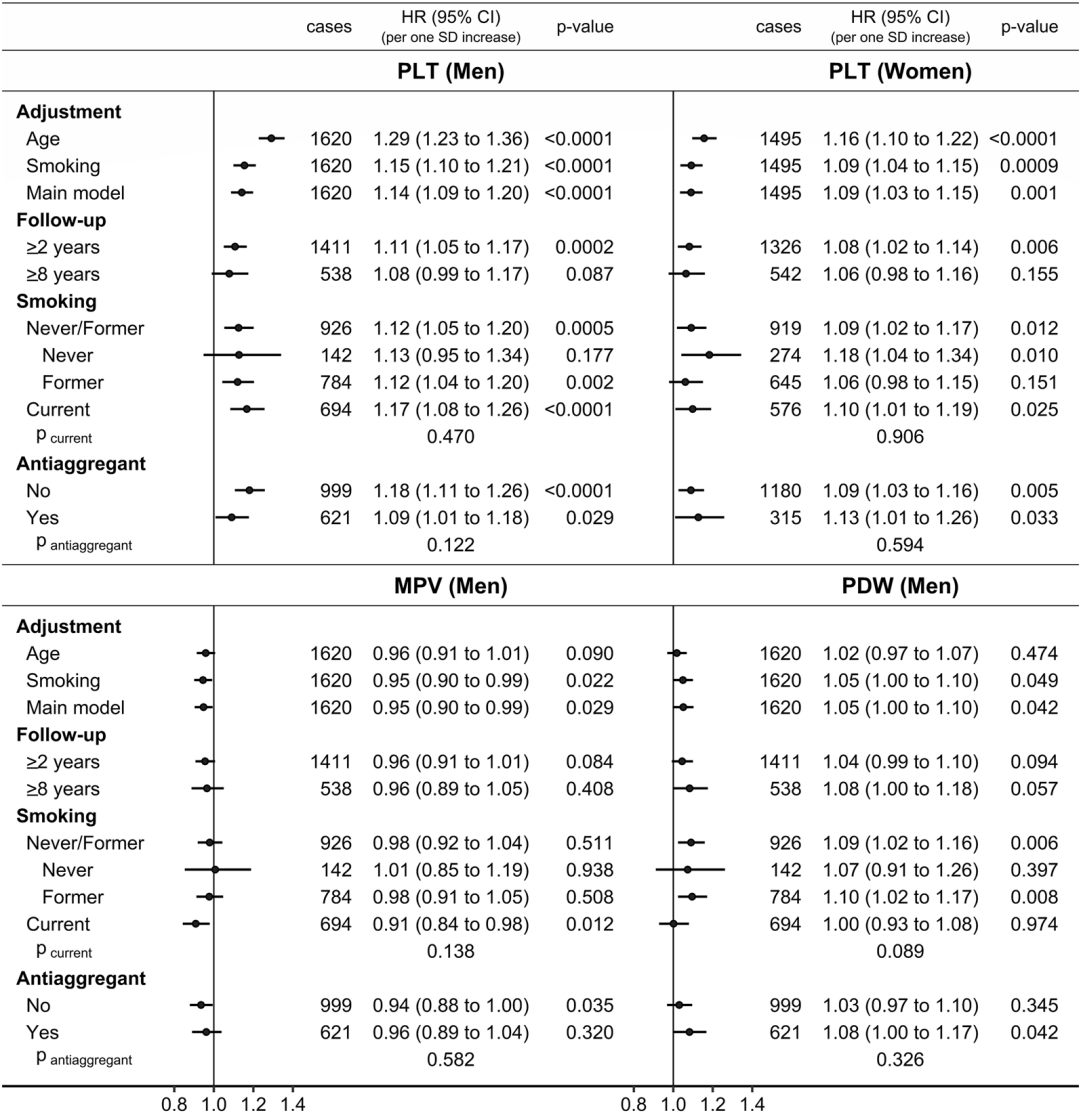
Associations of platelet parameters (continuous scale) with lung cancer risk: sensitivity analyses. *Cases* number of lung cancer cases per model, *CI* confidence interval, *HR* hazard ratio, *MPV* mean platelet volume, *p-value* Wald test for the individual term, *PDW* platelet distribution width, *PLT* platelet count, *SD* standard deviation. Cox proportional hazards models with exposure either PLT, MPV, or PDW (sex-specific z-scores, value minus mean divided by standard deviation after log-transformation), with the following stratifications, adjustments, and follow-up times: Age—stratified by age at recruitment, with follow-up from recruitment and no adjustment. Smoking—like “Age”, additionally adjusted for smoking status and intensity. Main model—stratified by age at recruitment, region, and in women, menopausal status and hormone replacement therapy use, and adjusted for body mass index (BMI), height, recent weight change, smoking status and intensity, alcohol consumption, physical activity, Townsend deprivation index, family history of cancer, time of blood collection, fasting time, diabetes and use of lipid-lowering drugs, antihypertensive drugs, antiaggregant/anticoagulants, non-steroidal anti-inflammatory drugs, and paracetamol. Follow-up: ≥ 2 years / ≥ 8 years—like “Main model”, excluding participants with less than 2 or 8 years of follow-up and lagging the entry date with 2 or 8 years, correspondingly. Smoking: Never / Former / Current—like “Main model”, in groups according to smoking status (retaining the adjustment for smoking intensity and time since quit). Antiaggregant: No / Yes—like “Main model”, in groups according to antiaggregant/anticoagulant use. p _current_ & p _antiaggregant_—p-value comparing the association with PLT, MPV, or PDW between current and never/former smokers or between groups according to antiaggregant/anticoagulant use with the augmentation method of Lunn and McNeil [20]

The positive associations of PLT with lung cancer risk in men and women were stronger in the age-stratified unadjusted model and were partly attenuated after adjustment for smoking status and intensity, but with no material influence of additional stratification and adjustment for covariates (Fig. 2). The positive associations with PLT were retained to at least 8 years of follow-up, although with some attenuation, and were directionally consistent in all groups according to smoking status but, for men, were partly attenuated in antiaggregant/anticoagulant users.

The inverse association of MPV with lung cancer risk in men was influenced little by adjustment for smoking or covariates but was partly attenuated after 8 years of follow-up and in antiaggregant/anticoagulant users and was observed only in current smokers (HR = 0.91; 95%CI = 0.84–0.98) and not in never or former smokers (Fig. 2). On the contrary, the positive association with PDW in men was revealed only after adjustment for smoking and was retained to at least 8 years of follow-up. Moreover, it was observed only in never/former smokers (HR = 1.09; 95%CI = 1.02–1.16) and not in current smokers and was prominent in antiaggregant/anticoagulant users (HR = 1.08; 95%CI = 1.00–1.17) (Fig. 2).

**Fig. 3:**
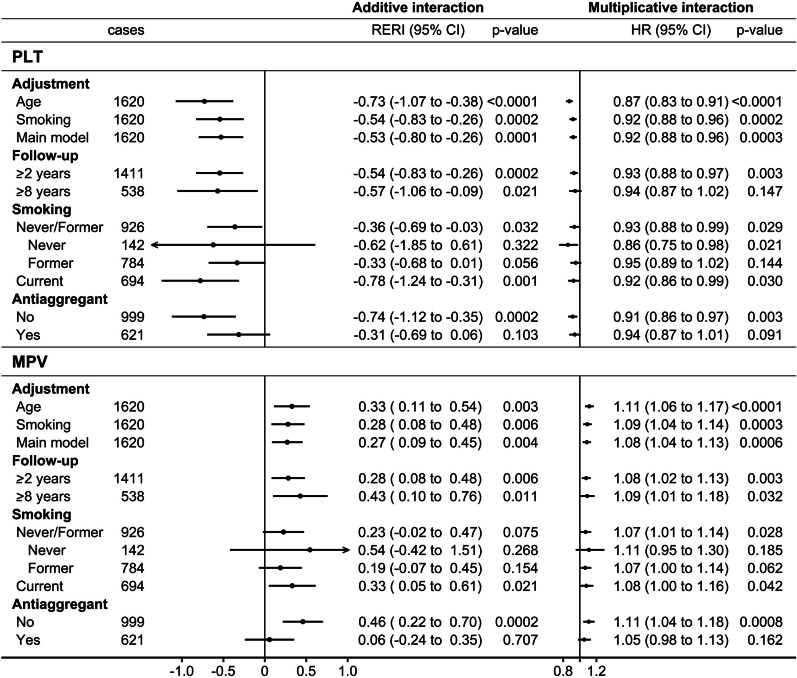
Additive and multiplicative interactions of platelet parameters with obesity: sensitivity analyses in men. *BMI* body mass index, *Cases* number of lung cancer cases, *CI* confidence interval, *HR* hazard ratio, *MPV* mean platelet volume, *p-value* p-value for RERI derived with the delta method or p-value from Wald test for the multiplicative interaction term, *PLT* platelet count, *RERI* relative excess risk from interaction (additive interaction). Cox proportional hazards models including a cross-classification with obese (additive interaction) or an interaction term with body mass index (BMI) on a continuous scale (multiplicative interaction) for either PLT or MPV in men with the following stratifications, adjustments, and follow-up times: Age—stratified by age at recruitment, with follow-up from recruitment and no adjustment. Smoking—like “Age”, additionally adjusted for smoking status and intensity. Main model—stratified by age at recruitment and region and adjusted for height, recent weight change, smoking status and intensity, alcohol consumption, physical activity, Townsend deprivation index, family history of cancer, time of blood collection, fasting time, diabetes, and use of lipid-lowering drugs, antihypertensive drugs, antiaggregant/anticoagulants, non-steroidal anti-inflammatory drugs, and paracetamol. Follow-up: ≥ 2/8 years—like “Main model”, excluding participants with less than 2 or 8 years of follow-up and lagging the entry date with 2 or 8 years, correspondingly. Smoking: Never / Former / Current—like “Main model”, in groups according to smoking status (retaining the adjustment for smoking intensity and time since quit). Antiaggregant: No / Yes—like “Main model”, in groups according to antiaggregant/anticoagulant use. Groups for cross-classifications (dichotomised to high/low) were defined as follows: BMI ≥ 30 kg/m^2^ (obese) or ≥ median (sex-specific) for PLT (234.0 men; 261.4 women) and MPV (9.17 men; 9.25 women)

The inverse interactions of PLT with BMI and the positive interactions of MPV with BMI were partly attenuated after adjustment for smoking but with no material influence of additional adjustment for covariates and were largely retained for at least 8 years of follow-up (Fig. 3). They remained directionally consistent in groups according to smoking status but were attenuated in antiaggregant/anticoagulant users.

Antiaggregant/anticoagulant users had higher lung cancer risk compared to non-users, independent of PLT, BMI, and covariates, but more prominently in men (HR = 1.20; 95%CI = 1.06–1.37) than in women (HR = 1.10; 95%CI = 0.95–1.27).

## Discussion

In this study, PLT was associated positively with lung cancer risk in women and men but showed consistent inverse additive and multiplicative interactions with BMI only in men. Also only in men, MPV was associated inversely with lung cancer risk and showed positive additive and multiplicative interactions with BMI, while PDW was associated weakly positively, with no evidence for interactions with obesity.

Our findings corroborate previous prospective studies reporting positive associations of PLT with lung cancer risk within the year prior to diagnosis [4, 27] and for at least ten years prior to diagnosis [6]. Given that lung cancer has unfavourable prognosis, with a global mortality-to-incidence ratio as high as 0.82 [28], longer-term prospective associations with PLT are compatible with a mechanistic involvement of platelets in lung cancer development. A causal association is further supported by positive associations of genetically predicted PLT, which has high genetic heritability [29], with lung cancer risk [30]. To our knowledge, however, there are no previous prospective studies to be able to compare the prospective associations of MPV and PDW with lung cancer risk described in our study. Several case–control studies have previously reported higher MPV at lung cancer diagnosis [14], in contrast to our findings, but a case–control study examining patients with advanced lung cancer has reported lower MPV at diagnosis, in addition to higher platelet count [31]. Although prospective associations may be retained to cancer diagnosis, studies recruiting cases and controls at cancer diagnosis would also reflect cancer-related changes and could thus be influenced by reverse causality, hence potentially explaining the differences between previous studies and our findings. Reports of poor prognosis for lower MPV measured at diagnosis [31, 32] may be more relevant to our findings because these are based on prospective studies and reflect lung cancer progression, which may involve pathways relevant to lung cancer development. Cancer survival, however, is dependent on comorbidities related to platelet activity, as well as on cancer progression and metastasis, and a large meta-analysis has found little evidence for association of MPV measured at diagnosis with overall survival [33]. Our findings are compatible with a small scale study reporting higher PDW at lung cancer diagnosis [15], but only for men. The retention of the positive association with PDW to at least 8 years of follow-up in our study suggests that this more likely reflects the influence of platelets on lung cancer development, rather than reverse causality.

A plausible mechanism linking PLT to lung cancer development would be an inflammation-related platelet increase, as platelets are involved in immuno-inflammatory responses [34] and PLT is high in chronic inflammatory conditions [35, 36]. Inflammatory markers have, indeed, been associated with higher lung cancer risk, more commonly when measured within the years close to diagnosis and in smokers [37], but also further away from diagnosis [38] and in never smokers [39]. Platelets contribute to cancer-associated inflammation by regulating the migration of haematopoietic and immune cells towards the tumour cite and facilitate cancer progression and metastasis by enabling thrombosis and the formation of neutrophil extracellular traps, which protect cancer cells [40]. Platelet-derived factors are also involved in immunomodulation, as is the case with TREM-like transcript 1 (TLT-1) protein, which is higher in platelets from patients with lung cancer and promotes cancer progression via suppression of CD8 T-cells [41]. The lung may be a particularly vulnerable organ to platelet action, as platelets are released in the lung from circulating megakaryocytes [3]. Although the stronger association with PLT closer to lung cancer diagnosis, described in our and in previous studies [5, 6], indicates an additional cancer-related PLT increase, the cancer is likely to promote an already operational inflammatory pathway.

Notably, the inverse association of MPV with lung cancer risk in men was observed in the same BMI categories (normal weight and overweight) as the positive association with PLT. This is consistent with the inverse correlation between PLT and MPV, which we have previously shown in UK Biobank for a restricted dataset excluding participants with cardiometabolic conditions [10] and have confirmed in this study for the unrestricted UK Biobank dataset. A potential explanation for an inverse association with MPV coupled to a positive association with PLT would be a trade-off between platelet size and count related to platelet formation, as immature proplatelet intermediates are larger particles and split into two smaller-size platelets in the process of maturation [13]. Mutations in megakaryocyte cytoskeleton proteins are, indeed, accompanied with large platelet size coupled to low platelet number [42]. Therefore, large platelet size may reflect more immature and potentially dysfunctional platelets and, hence, a lower risk of lung cancer development. Large platelet size, however, could also indicate platelet activation, because large platelets are more responsive to stimulation and less susceptible to suppression by aspirin [43] and MPV is associated positively with markers of platelet activation [44]. As platelet activation would result in a positive rather than an inverse association with lung cancer risk, a suggestion has previously been offered that larger activated platelets are engaged in thrombotic events, leaving only smaller platelets in the circulation of patients with lung cancer [31].

Although MPV and PDW are associated positively with each other in UK Biobank ([10] and this study) and both are higher in conditions involving platelet activation [45], they were associated with lung cancer risk in opposite directions (inverse for MPV, positive for PDW) and in different groups according to smoking status (current smokers for MPV, never/former smokers for PDW) and antiaggregant/anticoagulant use (non-users for MPV, users for PDW). This suggests that MPV and PDW reflect different underlying mechanisms linking platelets to lung cancer development, with larger MPV more likely reflecting lower lung cancer risk due to platelet immaturity and wider PDW more likely reflecting higher lung cancer risk due to platelet activation. One example of a mechanism of platelet activation differentially affecting MPV and PDW is DNA methylation of platelet-endothelial aggregation receptor 1 (PEAR-1), which is associated positively with PDW but not with MPV [46]. It is unknown, however, whether PEAR-1 is related to lung cancer risk.

PLT and MPV interacted with BMI in opposite directions in men, with obesity apparently hindering their associations with lung cancer risk, but potentially via different mechanisms. Thus, the inverse interaction of PLT with BMI is likely related to obesity contributing to non-alcoholic fatty liver disease (NAFLD) [47], which can lead to liver fibrosis, and this in turn can contribute to platelet destruction and removal of platelets from the circulation [48], as we have previously discussed in relation to the inverse association of BMI with PLT in UK Biobank men [10]. The positive interaction of MPV with BMI, on the other hand, is likely related to oestrogens, which are generated peripherally by adipose tissue aromatase [49], and are higher in obese UK Biobank men [24]. In accordance, oestrogens contribute to lung cancer development and progression [50], including in never smokers [51], and polymorphisms in the aromatase gene are associated with higher lung cancer risk [52]. Supporting a link of high-MPV with oestrogens, MPV is higher in women compared to men [53], oestrogen containing HRT increases MPV [54], tamoxifen (an oestrogen receptor modulator with oestrogenic effects outside the breast) also increases MPV [55], and oestradiol (either synthesised within megakaryocytes or extracellular) stimulates the formation of proplatelets, which are larger than mature platelets [56]. In addition, oestradiol can induce platelet aggregation via oestrogen receptor beta in men and may thus facilitate platelet action [57].

The associations of platelet parameters with lung cancer risk and their interactions with obesity showed sex differences, as previously did the associations of platelet parameters with obesity in UK Biobank [10]. This may be explained by the already higher PLT and higher platelet reactivity in women [58], which may limit additional influences from variations in PLT and MPV. Female sex and oestrogens also appear protective against NAFLD related fibrosis [59] and thrombopoietin levels are higher in obese women [60], potentially resulting in stimulated thrombopoiesis, which would explain the positive association of BMI with PLT [10] and may be preventing an inverse interaction of PLT with obesity in UK Biobank women.

Despite the detailed adjustment for smoking status and intensity, some residual confounding from smoking may have remained in the positive association of PLT with lung cancer risk, as PLT is higher in smokers [26]. We did not find, however, evidence for heterogeneity of the positive association with PLT between smoking status categories, although lung cancer cases were fewer in never smokers and power was limited, especially for men. The inverse association with MPV, on the other hand, could not reflect residual confounding from smoking because smoking is associated with higher MPV [26]. While oestrogens contribute to higher MPV and higher lung cancer risk, as outlined above, tobacco smoke components contribute to oestrogen inactivation [61], which may explain why the inverse association of MPV with lung cancer risk was relevant specifically to current smokers, with large MPV potentially reflecting platelet immaturity rather than platelet activation at lower oestrogen levels. Although smoking is associated with higher PDW [26], the positive association with PDW is less likely to be influenced by residual confounding from smoking, because it was not observed in current smokers, possibly because the PDW-related pathways are already activated in current smokers.

Although there is interest in using aspirin for lung cancer prevention [62], it would be hard to separate in observational settings aspirin use from the conditions requiring aspirin use. Thus, lung cancer risk was higher in antiaggregant/anticoagulant users in our study, which is compatible with higher lung cancer risk described for cardiovascular conditions [63, 64]. Therefore, the attenuation of the associations and interactions with PLT and MPV in antiaggregant/anticoagulant users most likely corresponds to already higher platelet activity in this group, with little possibility left for further influence of variations in PLT and MPV. On the other hand, PDW was associated positively with lung cancer risk only in antiaggregant/anticoagulant users and may thus reflect the extent of platelet activation in this group. Although our study cannot answer the question whether aspirin use modifies lung cancer risk, we have shown modification of the associations of platelet parameters with lung cancer risk by obesity related factors, at least in men, which supports the possibility for modifying lung cancer risk by modifying PLT and platelet action.

A major strength of our study is the prospective cohort design with available platelet measurements and a sizable number of incident lung cancer cases, which permitted examining cross-classifications. Anthropometric measurements, performed by trained personnel and according to standardised protocols, avoided bias from self-reporting. Information for major lifestyle factors (including smoking intensity and time since quit) and drug use permitted adjustment and minimisation of confounding.

A clear limitation of our study is the lack of information about platelet activation or about blood clotting factors, so we were unable to assess platelet function and thrombosis. We were also unable to examine thrombopoiesis and platelet maturity. Our project did not have access to the information for air pollution available in UK Biobank, as examining this was beyond the scope of our project but merits investigation in future studies because air pollution is associated with platelet activation [65], as well as with higher risk of lung cancer [66]. Some residual confounding from smoking is possible for the positive association with PLT, as smoking was the most influential covariate. The number of lung cancer cases was insufficient to assess differences in the interaction patterns between lung cancer subtypes, although no major differences have been reported for the positive associations with PLT between lung cancer subtypes [5, 30]. Exposures and confounders were measured only once, at cohort recruitment, so changes during follow-up could not be accounted for. UK Biobank participants have healthier lifestyle compared to the general population [67] and mainly have white ethnic background, preventing investigation of ethnic differences. Last, the reported associations may not be causal, due to the observational nature of the study.

## Conclusions

In men, PLT was associated positively and MPV inversely with lung cancer risk and obesity appeared to hinder these associations, possibly via platelet destruction due to obesity related liver fibrosis for PLT and via oestrogen related platelet activation for MPV. In women, only PLT was associated positively with lung cancer risk, with little evidence for interaction with obesity.

### Supplementary Information

**Additional file 1: Table S1.** Flow chart of study participants. **Table S2.** Characteristics of study participants.

## Data Availability

The dataset analysed in the current study was used under license and cannot be made freely available in a public repository or obtained from the authors due to restrictions related to privacy regulations and informed consent of the participants. Access to the data, however, can be obtained by *bona fide* researchers from UK Biobank, subject to approval of the research project and a material transfer agreement. For information on how to gain access to UK Biobank data, please follow the instructions at https://www.ukbiobank.ac.uk/enable-your-research. Further queries related to the data could be addressed to the corresponding author Dr Sofia Christakoudi: s.christakoudi@imperial.ac.uk.

## Abbreviations

BMI: Body mass index
CI: Confidence interval
HR: Hazard ratio
HRT: Hormone replacement therapy
ICD10: 10Th revision of the International Statistical Classification of Diseases
MPV: Mean platelet volume
PDW: Platelet distribution width
PLT: Platelet count
SD: Standard deviation
UK: United Kingdom
