# Supplementary material for: Interactions of platelets with obesity in relation to lung cancer risk in the UK Biobank cohort
Source: Respir Res. 2023 Oct 17;24:249. doi: 10.1186/s12931-023-02561-9 (PMC10580651; doi:10.1186/s12931-023-02561-9)
Supplement: Supplementary file 1 — Additional file 1: Table S1. Flow chart of study participants. Table S2. Characteristics of study participants. [file 12931_2023_2561_MOESM1_ESM.docx]

Interactions of platelets with obesity in relation to lung cancer risk in the UK Biobank cohort

Sofia Christakoudi, Konstantinos K. Tsilidis, Evangelos Evangelou, Elio Riboli

Additional Methods

[Selection of non-smoking covariates 2](#_Toc145432866)

[Rationale for including BMI and height in the same model 2](#_Toc145432867)

[Definition of covariates 3](#_Toc145432868)

**Additional Tables**

[Table S1 Flow chart of study participants 8](#_Toc145432869)

[Table S2 Characteristics of study participants 9](#_Toc145432870)

[References………………………………………………………………………………………………….12](#_Toc145432871)

## Selection of non-smoking covariates

Non-smoking covariates were selected *a priori*, based on previous reports for associations with platelet parameters and lung cancer risk, as explained below. The adjustment for drug use aimed to account for exogenous influences on metabolic and inflammatory conditions, thrombosis, and liver fat accumulation and function, which can affect platelet parameters and platelet activity.

Alcohol consumption can contribute to liver fat accumulation and fibrosis [68] and the inverse association of platelet count (PLT) with body mass index (BMI), which we have previously described for UK Biobank men, was strongest for daily alcohol consumption [10]. At the same time, lower lung cancer risk has been described in light and moderate drinkers compared to non-drinkers among never smokers [69]. PLT and platelet aggregation increase after strenuous physical exercise [70], but there is evidence that higher long term physical activity is associated with lower lung cancer risk [71]. Low socioeconomic status (proxied in our study by Townsend deprivation index) is associated with less healthy lifestyle and higher lung cancer risk [72]. Patients with type 2 diabetes mellitus or impaired fasting glucose have higher PLT, higher mean platelet volume (MPV), wider platelet distribution width (PDW) [73] and, at least for women, higher lung cancer risk [74]. We have adjusted for use of antihypertensive drugs, as indicative of consistent, clinically diagnosed hypertension. Systolic and diastolic blood pressure are associated positively with PLT and PDW [75], and systolic blood pressure is also associated positively with lung cancer risk [76]. Lipid-lowering drugs, specifically statins, can reduce liver fat accumulation and the risk of developing non-alcoholic fatty liver disease (NAFLD) [77]. Paracetamol has substantial hepatotoxicity [78] and regular paracetamol use has been associated with higher lung cancer risk [79]. Platelets are key participants in immuno-inflammation [34], and their function is likely to be affected by nonsteroidal anti-inflammatory drugs (NSAID), antiaggregants, and anticoagulants. Blood samples in UK Biobank were collected throughout the day irrespective of fasting status and we have included adjustment for time of blood collection, to account for diurnal variations in platelet parameters [80], and for fasting time, to account for post-prandial changes [81]. We have stratified the models by menopausal status in women, because the positive association of BMI with PLT, that we have previously described for UK Biobank women, was weaker in post-menopausal compared to premenopausal women [10]. We have also stratified by use of hormone replacement therapy (HRT) because sex-steroids are likely to contribute to differences between women and men and between pre-menopausal and post-menopausal women and HRT has been associated with lower lung cancer risk [82]. Last, we have included as covariate weight change within the year preceding recruitment, to account for recent BMI dynamics.

## Rationale for including BMI and height in the same model

BMI (Quételet index) has been developed as an index of relative weight to evaluate weight independent of height, so BMI is designed to be uncorrelated with height. The power coefficient for height in the formula for BMI has been established empirically, based on data collected in the 19^th^ century, and indicates that weight increases proportionally to the square of height [83]. As BMI is based on a model which was fitted in a 19^th^ century dataset, this may not be calibrated well in contemporary datasets. Indeed, in UK Biobank, BMI is correlated weakly inversely with height (correlation coefficients -0.06 for men and -0.11 for women [17]). Including height as an adjustment variable in the model aims to account for the weak residual correlation of BMI with height, and thus to ensure that BMI truly reflects associations independent of height, as intended.

We have previously shown that such adjustment approach is able to compensate for residual correlations in relation to another allometric index, hip index [17]. In analogy to BMI, hip index has been created to evaluate hip circumference independent of BMI and height using data from the National Health and Nutrition Examination Survey (NHANES) [84]. However, although hip index calculated with power coefficients based on NHANES (HI_NHANES_), i.e. it is calibrated in NHANES and thus is uncorrelated with BMI and height in NHANES, it was substantially inversely correlated with BMI in UK Biobank men (corelation coefficient -0.31 [17]). Nevertheless, estimates for cancer risk obtained from models adjusting HI_NHANES_ for BMI and height showed no material difference to risk estimates for hip index calculated with power coefficients based on UK Biobank (HI_UKB_), which was uncorrelated with BMI and height in UK Biobank because it was re-calibrated in the UK Biobank dataset [17].

## Definition of covariates

The following variables were defined as previously described in [17]: age at recruitment (5-year categories for stratification of survival models), region of the assessment centre (London, North-West, North-East, Yorkshire and Humber, West Midlands, East Midlands, South-East, South-West, Wales, Scotland), weight change within the year preceding recruitment (weight loss, stable weight, weight gain), alcohol consumption (≤3 times/month, ≤4 times/week, daily), physical activity (less active, moderately active, very active), Townsend deprivation index (sex-specific quintile cut-offs: -3.985, -2.870, -1.487, 1.088 for men; -3.979, -2.865, -1.532, 0.925 for women). HRT use (never, former, current) was defined as previously described in [ref. 24], taking into account self-reported current medication (listed in Supplementary Table S1 of [24]) The following variables were defined as previously described in [25]: menopausal status (pre-menopausal (excluding women with hysterectomy), post-menopausal (including women with bilateral oophorectomy), unknown); fasting time (0-2 hours, 3-4 hours, ≥5 hours); time of blood collection (morning <12:00, afternoon 12:00 to <16:00, evening ≥16:00 o’clock); diabetes status (yes/no); antihypertensive drugs (yes/no) (see further details in Supplementary Methods of [25] and lists of medications in Supplementary Table S1 of [25]). Lipid lowering drugs use (yes/no) was also defined as in [ref. 25], but additionally reclassifying as “Yes” based on the following codes from Fields [20003-0.0…47] “*Treatment/medication code*” (1140865576 cholestyramine; 1140909780 colestyramine; 1141157416 cholestyramine product). Paracetamol use (yes/no) was also defined as in [25], but additionally reclassifying as “Yes” based on the following code from Fields [20003-0.0…47] “*Treatment/medication code*” (1140871968 isometheptene mucate+paracetamol 65mg/325mg capsule). Missing values were assigned the median sex-specific category as follows: weight change (stable weight), alcohol consumption (≤4 times/week), physical activity (moderately active), Townsend deprivation index (third quintile), HRT use (never), fasting time (3-4 hours), time of blood collection (afternoon); diabetes status (no); antihypertensive drugs (no); lipid-lowering drugs (no); paracetamol (no).

Family history of cancer was defined using three sets of fields as in [17]: Fields [20107-0.0/9] “*Illness of father*”, Fields [20110-0.0/10] “*Illness of mother*”, and Fields [20111-0.0/11] “*Illness of siblings*”. Category “Breast/prostate/bowel” was based on Answers: 5 “Breast cancer”, OR 13 “Prostate cancer”, OR 4 “Bowel cancer”, to any of the three sets of fields. Category “Lung cancer” was based on Answer: 3 “Lung cancer” to any of the three sets of fields. Category “No cancer” included all remaining participants.

Smoking status (used for cross-classification and stratification) was defined with three categories as follows: Category “Current smoker” – was based on Field [1239-0.0] “*Current tobacco smoking*”; Question: "*Do you smoke tobacco now?*"; Answer 1: “Yes, on most or all days” OR Answer 2: “Only occasionally”; Category “Former smoker” – was based on Field [1249-0.0] “*Past tobacco smoking*”; Question: "*In the past, how often have you smoked tobacco?*"; Answer 1: “Smoked on most or all days” OR Answer 2: “Smoked occasionally”, when the answer to Filed [1239-0.0] was not 1 or 2; Category “Never smoker” – was based on Field [1249-0.0] Answer 3: “Just tried once or twice” OR Answer 4: “I have never smoked”, when the answer to [Filed 1239-0.0] was not 1 or 2. Missing values were assigned the median sex-specific category “Never smoked” for women and “Former smoker” for men.

Smoking status and intensity (used for adjustment) was defined using smoking status as follows: Category “Never smoked” was divided based on Field [1249-0.0] in two categories: “Never smoked” – based on Answer 4: “I have never smoked“ and “Just tried” – based on Answer 3: “Just tried once or twice”; Category “Former smoker” was divided in four categories: “Former occasional” – based on Field [1249-0.0] Answer 2: “Only occasionally”; “Former regular: quit≥20 years”; “Former regular: quit ≥10 years”; and “Former regular: quit <10 years” – based on time since quit calculated as the difference between Field [2897-0.0] “Age stopped smoking” and age at recruitment; Category “Current smoker” was divided in three groups: “Current occasional” – based on Field [1239-0.0] Answer 2: “Only occasionally”; “Current regular: ≤10 cigarettes” and ”Current regular: >10 cigarettes” – based on the number of cigarettes per day from Field [3456-0.0] “Number of cigarettes currently smoked daily (current cigarette smokers)”; Question: "In the past, how often have you smoked tobacco?". Missing values were assigned the median sex-specific category per smoking status: “Never smoked” for both sexes; “Former regular: quit ≥20 years” for both sexes; “Current regular: ≤10 cigarettes” for women and “Current regular: >10 cigarettes” for men.

Antiaggregant / Anticoagulant use was based on Fields [6154-0.1/5] “*Medication for pain relief, constipation, heartburn*”; Question: “*Do you regularly take any of the following? (You can select more than one answer)*” Answer 1: “Aspirin” for category “Yes”. Category “No” was defined as any of the following Answers: 2 “Ibuprofen”, 3 “Paracetamol”, 4 “Ranitidine”, 5 “Omeprazole”, 6 “Laxatives” OR -7 “None of the above”, and information for the remaining participants was considered missing. We re-classified to category “Yes” participants with self-reported antiaggregant use in Fields [20003-0/47] “Treatment/ medication code” with the following codes (UK Biobank Coding 4):

1140856412 norgesic tablet

1140861776 antiplatelet drug

1140861778 dipyridamole

1140861780 persantin 25mg tablet

1140861790 cerebrovase 25mg tablet

1140861800 platet 100mg effervescent tablet

1140861804 angettes 75mg tablet

1140861806 aspirin 75mg tablet

1140861808 disprin cv 100mg m/r tablet

1140864860 nu-seals aspirin 75mg e/c tablet

1140868226 aspirin

1140868258 aspav dispersible tablet

1140868282 aspirin+methocarbamol 325mg/400mg tablet

1140872040 aspirin+metoclopramide 325mg/5mg effervescent tablet

1140882108 aspirin+cyclizine hydrochloride 500mg/25mg tablet

1140882190 aspirin+glycine 500mg/133mg dispersible tablet

1140882268 aspirin+codeine 300mg/8mg tablet

1140882392 aspirin+codeine

1140909772 acetylsalicylic acid

1140909890 sulfinpyrazone

1140911756 askit powder

1141163138 aspirin+papaveretum 500mg/7.71mg dispersible tablet

1141164044 isosorbide mononitrate+aspirin

1141167844 dipyridamole+aspirin

1141167848 asasantin retard m/r capsule

1141168318 clopidogrel

1141168322 plavix 75mg tablet

We additionally reclassified to category “Yes” participants with self-reported anticoagulant use in Fields [20003-0/47] with the following codes:

1140861506 calciparine 5000iu/0.2ml prefilled syringe

1140861568 minihep calcium 5000iu/0.2ml injection

1140861574 uniparin-ca 5000iu/0.2ml prefilled syringe

1140861578 monoparin-ca 5000iu/0.2ml injection

1140861584 fragmin 10,000iu/1ml injection

1140861588 enoxaparin

1140861594 clexane 20mg/0.2ml prefilled syringe

1140861602 innohep 5000iu/0.5ml injection amp

1140861604 logiparin 2500iu/0.21ml prefilled syringe

1140861696 nicoumalone

1140861698 sinthrome 1mg tablet

1140861702 phenindione

1140861704 dindevan 10mg tablet

1140864956 subcutaneous heparin

1140877958 heparinoid+salicylic acid 0.2%/2% cream

1140877960 heparinoid+salicylic acid 0.2%/2% gel

1140881842 heparin

1140888204 dalteparin

1140888206 tinzaparin

1140888266 warfarin

1140909770 acenocoumarol

1140910832 sodium warfarin

1140926360 alphaparin 3000iu/0.3ml prefilled syringe

1140926444 certoparin

1141171364 reviparin

1141171374 clivarine 1432iu/0.25ml prefilled syringe

1141189054 bemiparin

Missing values were assigned the median sex-specific category “No” for both sexes.

Nonsteroidal anti-inflammatory drugs (NSAID) use was based on Fields [6154-0.1/5] “*Medication for pain relief, constipation, heartburn*”; Question: “*Do you regularly take any of the following? (You can select more than one answer)*” Answer 2: “Ibuprofen” for category “Yes”. Category “No” was defined as any of the following Answers: 1 “Aspirin”, 3 “Paracetamol”, 4 “Ranitidine”, 5 “Omeprazole”, 6 “Laxatives” OR -7 “None of the above”, and information for the remaining participants was considered missing. We re-classified to category “Yes” participants with self-reported NSAID use in Fields [20003-0.0…47] “*Treatment/medication code*” for the following codes (UK Biobank Coding 4):

| 1140868336 | synflex 275mg tablet | 1140871564 | rimoxyn 250mg tablet |
| --- | --- | --- | --- |
| 1140871080 | benorylate | 1140871568 | nycopren 250mg e/c tablet |
| 1140871082 | benoral 750mg tablet | 1140871582 | pirozip 10 capsule |
| 1140871092 | salsalate | 1140871590 | flamatrol 10mg capsule |
| 1140871094 | disalcid 500mg capsule | 1140871604 | sulindac |
| 1140871100 | azapropazone | 1140871606 | clinoril 100mg tablet |
| 1140871102 | rheumox 300mg capsule | 1140871614 | tiaprofenic acid |
| 1140871168 | voltarol 25mg e/c tablet | 1140871616 | surgam 200mg tablet |
| 1140871174 | voltarol 100mg suppository | 1140871628 | prosaid 250mg tablet |
| 1140871180 | rhumalgan 25mg e/c tablet | 1140871638 | napratec tablet combination pack |
| 1140871188 | etodolac | 1140871654 | phenylbutazone product |
| 1140871202 | fenbufen | 1140871660 | butacote 100mg e/c tablet |
| 1140871206 | lederfen 300mg tablet | 1140871662 | butazone 100mg tablet |
| 1140871218 | fenbuzip 300mg tablet | 1140871666 | piroxicam |
| 1140871226 | fenoprofen | 1140871672 | feldene 10mg capsule |
| 1140871228 | fenopron 300mg tablet | 1140875336 | nabumetone |
| 1140871236 | flurbiprofen | 1140875338 | relifex 500mg tablet |
| 1140871238 | froben 50mg tablet | 1140875346 | tenoxicam |
| 1140871248 | volraman 25mg e/c tablet | 1140875546 | piroxicam 0.5% gel |
| 1140871256 | valenac 25mg e/c tablet | 1140875630 | movelat cream |
| 1140871260 | diclozip-25 e/c tablet | 1140875632 | movelat gel |
| 1140871266 | arthrotec tablet | 1140875640 | traxam gel |
| 1140871274 | isclofen 50mg e/c tablet | 1140875642 | ketoprofen 2.5% gel |
| 1140871276 | flamrase 25mg e/c tablet | 1140877880 | fepron 600mg tablet |
| 1140871282 | diflunisal | 1140878030 | ibuprofen+codeine phosphate |
| 1140871284 | dolobid 250mg tablet | 1140878036 | diclofenac sodium+misoprostol |
| 1140871310 | ibuprofen | 1140881612 | naproxen+misoprostol |
| 1140871320 | arthrofen 200 tablet | 1140883812 | parfenac 5% cream |
| 1140871336 | indomethacin | 1140884488 | diclofenac |
| 1140871344 | artracin 25mg capsule | 1140884498 | felbinac |
| 1140871348 | imbrilon 25mg capsule | 1140910496 | propionic acid-ibuprofen |
| 1140871354 | indocid 25mg capsule | 1140910686 | hydroxyphenylbutazone |
| 1140871360 | flexin-25 continus m/r tablet | 1140911748 | ibuprofen+menthol 5%/3% gel |
| 1140871370 | apsifen 200mg tablet | 1140911750 | deep relief ibuprofen gel |
| 1140871374 | brufen 200mg tablet | 1140911754 | anadin tablet |
| 1140871386 | ebufac 200mg tablet | 1140925806 | aceclofenac |
| 1140871388 | cuprofen 200mg tablet | 1140925808 | preservex 100mg tablet |
| 1140871392 | isisfen 400mg tablet | 1140926732 | meloxicam |
| 1140871394 | fenbid 300mg spansule | 1141145722 | slofenac sr 75mg m/r tablet |
| 1140871396 | lidifen 200mg tablet | 1141149110 | cuprofen 5% gel |
| 1140871402 | codafen continus m/r tablet | 1141153134 | anadin ibuprofen 200mg tablet |
| 1140871404 | junifen 100mg/5ml s/f suspension | 1141157412 | ibuprofen product |
| 1140871406 | motrin 200mg tablet | 1141157452 | indomethacin product |
| 1140871408 | ibumed 400mg tablet | 1141164746 | dexketoprofen |
| 1140871416 | rimafen 200mg tablet | 1141164750 | keral 25mg tablet |
| 1140871430 | rimacid 25mg capsule | 1141165574 | galprofen 100mg/5ml oral suspension |
| 1140871434 | indomod 25mg m/r capsule | 1141165754 | librofem 200mg tablet |
| 1140871442 | indomax 25 capsule | 1141169526 | piroxicam-beta-cyclodextrin |
| 1140871454 | contraflam 250mg capsule | 1141169530 | brexidol 20mg tablet |
| 1140871462 | naproxen | 1141182674 | fenactol 25mg e/c tablet |
| 1140871468 | laraflex 250mg tablet | 1141182708 | ipocol 400mg e/c tablet |
| 1140871472 | naprosyn 250mg tablet | 1141182754 | piroxicam-betadex |
| 1140871482 | rheuflex-250 tablet | 1141184156 | lornoxicam |
| 1140871484 | valrox 250mg tablet | 1141184162 | xefo 4mg tablet |
| 1140871490 | pranoxen continus 375mg m/r tablet | 1141184290 | vioxxacute 25mg tablet |
| 1140871506 | ketoprofen | 1141184292 | vioxxacute 50mg tablet |
| 1140871516 | orudis 50mg capsule | 1141184546 | ibuprofen+pseudoephedrine hydrochloride |
| 1140871522 | oruvail 100 m/r capsule | 1141187776 | nurofen 200mg tablet |
| 1140871528 | ketovail 100mg m/r capsule | 1141190952 | cuprofen plus tablet |
| 1140871532 | ketonal 50mg capsule | 1141191742 | calprofen 100mg/5ml s/f oral suspension |
| 1140871542 | mefenamic acid | 1141194296 | lemsip flu 12hr ibuprofen+pseudoephedrine capsule |
| 1140871546 | ponstan 250mg capsule | 1141200748 | care ibuprofen 10% gel |
| 1140871556 | arthroxen 250mg tablet |  |  |

We additionally reclassified to category “Yes” participants with self-reported aminosalicylates use in Fields [20003-0/47] with the following codes:

1140865578 mesalazine

1140865668 sulphasalazine

1140865670 salazopyrin 500mg tablet

1140879502 olsalazine

1140909702 sulfasalazine

1140909776 etamsylate

1140910598 5asa - mesalazine

1140910600 aminosalicylic acid

1140910678 azodisalicylic acid

1140910680 disodium azodisalicylic acid

Missing values were assigned the median sex-specific category “No” for both sexes.

## Table S1 Flow chart of study participants

|  | **Exclusions Total** | **Men** | **Women** |
| --- | --- | --- | --- |
|  | Total (excluding withdrawals up to the time of analysis): 502,369 | 229,068 | 273,301 |
| 1. | Ethnic background (restricted to self-reported white) ^a^ | 13,862 | 15,935 |
| 2. | Anthropometric measurements missing or extreme ^b^ | 2373 | 4758 |
| 3. | Genetic & self-reported sex mismatch or sex chromosome aneuploidy ^a^ | 396 | 386 |
| 4. | Age restrictions (age <40 or >70 years) | 9 | 2 |
| 5. | Pregnant or unknown at recruitment ^a^ | 0 | 105 |
| 6. | Prevalent cancer at recruitment ^a^ | 12,189 | 21,857 |
| 7. | Antihemorrhagic agents ^c^ | 14 | 582 |
| 8. | Platelet parameters missing | 7870 | 10,915 |
|  | Total excluded (%): 91,253 | 36,713 | 54,540 |
|  | (18.2) | (16.0) | (20.0) |
|  | **Total included: 411,116** | **192,355** | **218,761** |

The exclusion criteria were applied sequentially in the displayed order, counting each excluded individual only once.

**^a^** – for UK Biobank Field names, definition of variables, and definition of prevalent cancer cases see Supplementary Methods in [17].

**^b^** – missing anthropometric measurements; height <130 cm; waist circumference <50 or >160 cm; body mass index (BMI) <18.5 or ≥45 kg/m^2^. Field names for waist and hip circumferences, weight, and height are listed in Supplementary Methods of [17].

**^c^** – self-reported use of medications from Fields [20003-0/47] “Treatment/ medication code” with the following codes:

| 1140861766 | ethamsylate |
| --- | --- |
| 1140861832 | tranexamic acid |
| 1140861834 | cyklokapron 500mg tablet |

### Table S2 Characteristics of study participants

|  | **MEN** | **WOMEN** |
| --- | --- | --- |
| Cohort | 192,355 | 218,761 |
| **Anthropometry**: mean (SD) |  |  |
| Height: cm | 175.9 (6.8) | 162.6 (6.2) |
| Weight: kg | 86.0 (13.7) | 71.2 (13.1) |
| Waist circumference: cm | 96.9 (11.0) | 84.3 (12.0) |
| Hip circumference: cm | 103.4 (7.2) | 103.1 (9.7) |
| **Weight change**: n (%) |  |  |
| Weight loss | 27,864 (14.5) | 33,178 (15.2) |
| Stable weight | 118,401 (61.6) | 111,439 (50.9) |
| Weight gain | 42,842 (22.3) | 70,714 (32.3) |
| Missing | 3248 (1.7) | 3430 (1.6) |
| **Smoking status**: n (%) |  |  |
| Never smoked | 65,799 (34.2) | 95,457 (43.6) |
| Just tried | 27,728 (14.4) | 33,210 (15.2) |
| Former occasional | 21,000 (10.9) | 26,790 (12.2) |
| Former regular: quit ≥20 years | 27,427 (14.3) | 20,842 (9.5) |
| Former regular: quit ≥10 years | 12,130 (6.3) | 10,229 (4.7) |
| Former regular: quit <10 years | 14,018 (7.3) | 12,151 (5.6) |
| Former regular: quit missing | 202 (0.1) | 207 (0.1) |
| Current occasional | 6517 (3.4) | 4494 (2.1) |
| Current regular: ≤10 cigarettes/day | 4521 (2.4) | 6009 (2.7) |
| Current regular: >10 cigarettes/day | 11,437 (5.9) | 8556 (3.9) |
| Current regular: cigarettes/day missing | 949 (0.5) | 117 (0.1) |
| Missing | 627 (0.3) | 699 (0.3) |
| **Alcohol consumption**: n (%) |  |  |
| ≤3 times / month | 39,056 (20.3) | 76,439 (34.9) |
| ≤4 times / week | 102,751 (53.4) | 105,400 (48.2) |
| Daily | 50,399 (26.2) | 36,791 (16.8) |
| Missing | 149 (0.1) | 131 (0.1) |
| **Physical activity**: n (%) |  |  |
| Less active | 29,222 (15.2) | 36,442 (16.7) |
| Moderately active | 86,138 (44.8) | 114,041 (52.1) |
| Very active | 76,376 (39.7) | 67,498 (30.9) |
| Missing | 619 (0.3) | 780 (0.4) |
| **Family history**: n (%) |  |  |
| No cancer | 124,884 (64.9) | 140,467 (64.2) |
| Breast, bowel, prostate | 43,457 (22.6) | 50,070 (22.9) |
| Lung cancer | 24,014 (12.5) | 28,224 (12.9) |
| **Townsend index** |  |  |
| Median (IQR) | -2.27 (4.01) | -2.29 (3.85) |
| Missing: n (%) | 236 (0.1) | 246 (0.1) |
| **Time of sample**: n (%) |  |  |
| <12:00 | 51,153 (26.6) | 53,646 (24.5) |
| 12:00 to <16:00 | 74,304 (38.6) | 93,892 (42.9) |
| ≥16:00 | 66,797 (34.7) | 71,104 (32.5) |
| Missing | 101 (0.1) | 119 (0.1) |
| **Fasting time**: n (%) |  |  |
| 0-2 hours | 50,276 (26.1) | 57,135 (26.1) |
| 3-4 hours | 95,802 (49.8) | 114,942 (52.5) |
| ≥5 hours | 46,274 (24.1) | 46,678 (21.3) |
| Missing | 3 (<0.1) | 6 (<0.1) |
|  | **MEN** | **WOMEN** |
| **Diabetes**: n (%) |  |  |
| Yes | 12,241 (6.4) | 6567 (3.0) |
| Missing | 521 (0.3) | 384 (0.2) |
| **Lipid-lowering drugs use**: n (%) |  |  |
| Yes | 44,831 (23.3) | 27,520 (12.6) |
| Missing | 1459 (0.8) | 819 (0.4) |
| **Antihypertensive drugs use**: n (%) |  |  |
| Yes | 46,109 (24.0) | 36,804 (16.8) |
| Missing | 1638 (0.9) | 858 (0.4) |
| **Antiaggregant/anticoagulant use**: n (%) (**^#^**) |  |  |
| Aspirin | 36,558 (19.0) (91.1) | 21,784 (10.0) (92.8) |
| Antiaggregant (other than aspirin) | 895 (0.5) (2.2) | 516 (0.2) (2.2) |
| Anticoagulant only | 2685 (1.4) (6.7) | 1170 (0.5) (5.0) |
| Missing | 2046 (1.1) | 1867 (0.9) |
| **NSAID use**: n (%) |  |  |
| Yes | 29,796 (15.5) | 46,787 (21.4) |
| Missing | 2023 (1.1) | 1750 (0.8) |
| **Paracetamol use**: n (%) |  |  |
| Yes | 32,150 (16.7) | 57,921 (26.5) |
| Missing | 2159 (1.1) | 1875 (0.9) |
| **HRT use**: n (%) |  |  |
| Never |  | 132,371 (60.5) |
| Past |  | 66,692 (30.5) |
| Current |  | 19,203 (8.8) |
| Missing |  | 495 (0.2) |
| **Menopause**: n (%) |  |  |
| Pre-menopausal |  | 51,958 (23.8) |
| Post-menopausal |  | 142,294 (65.0) |
| Unknown |  | 24,509 (11.2) |
| **Menopause-HRT-use**: n (%) |  |  |
| Pre-menopausal |  | 51,958 (23.8) |
| Post/Unknown: Never |  | 82,872 (37.9) |
| Post/Unknown: Past |  | 65,756 (30.1) |
| Post/Unknown: Current |  | 18,175 (8.3) |
| **Age at recruitment:** n (%) |  |  |
| 40 to <45 years | 19,579 (10.2) | 21,912 (10.0) |
| 45 to <50 years | 24,415 (12.7) | 29,317 (13.4) |
| 50 to <55 years | 28,146 (14.6) | 34,547 (15.8) |
| 55 to <60 years | 34,248 (17.8) | 41,083 (18.8) |
| 60 to <65 years | 47,250 (24.6) | 53,560 (24.5) |
| 65 to 70 years | 38,717 (20.1) | 38,342 (17.5) |
| **Region**: n (%) |  |  |
| London | 22,043 (11.5) | 26,127 (11.9) |
| North-West | 30,310 (15.8) | 32,993 (15.1) |
| North-East | 23,689 (12.3) | 26,882 (12.3) |
| Yorkshire Humber | 29,319 (15.2) | 33,382 (15.3) |
| West Midlands | 17,904 (9.3) | 17,886 (8.2) |
| East Midlands | 13,358 (6.9) | 15,174 (6.9) |
| South-East | 16,857 (8.8) | 20,188 (9.2) |
| South-West | 16,755 (8.7) | 201,53 (9.2) |
| Wales | 8243 (4.3) | 9331 (4.3) |
| Scotland | 13,877 (7.2) | 16,645 (7.6) |

**HRT** – hormone replacement therapy; **IQR** – interquartile range; **n (%)** – number of participants (percentage from total per sex); **(^#^)** – percentage from total antiaggregant/anticoagulant users per sex; **NSAID** – non-steroidal anti-inflammatory drugs.

Comparisons between obesity categories and sexes were performed with unpaired-samples t-test for anthropometric measures, Wilcoxon rank sum (Mann-Whitney) test for Townsend deprivation index, and χ^2^-test for categorical variables (after imputation). All differences were significant at p<0.0001, except missing Townsend deprivation index (p>0.05).

For definition of medications see **Additional Methods**.

References

**References cited in the main document**:

10. Christakoudi S, Tsilidis KK, Evangelou E, Riboli E. Sex differences in the associations of body size and body shape with platelets in the UK Biobank cohort. *Biol Sex Differ*. 2023;14(1):12. doi:10.1186/s13293-023-00494-y.

17. Christakoudi S, Tsilidis KK, Evangelou E, Riboli E. A Body Shape Index (ABSI), hip index, and risk of cancer in the UK Biobank cohort. *Cancer Med*. 2021;10(16):5614-28. doi:10.1002/cam4.4097.

24. Christakoudi S, Riboli E, Evangelou E, Tsilidis KK. Associations of body shape phenotypes with sex steroids and their binding proteins in the UK Biobank cohort. *Sci Rep*. 2022;12(1):10774. doi:10.1038/s41598-022-14439-9.

25. Christakoudi, S., Riboli, E., Evangelou, E. & Tsilidis, K. K. Associations of body shape index (ABSI) and hip index with liver, metabolic, and inflammatory biomarkers in the UK Biobank cohort. *Sci Rep*. 2022;12(1):8812. doi:10.1038/s41598-022-12284-4.

34. Dib PRB, Quirino-Teixeira AC, Merij LB, Pinheiro MBM, Rozini SV, Andrade FB et al. Innate immune receptors in platelets and platelet-leukocyte interactions. *J Leukoc Biol*. 2020;108(4):1157-82. doi:10.1002/jlb.4mr0620-701r.

**Supplementary references**:

68. Jeon S, Carr R. Alcohol effects on hepatic lipid metabolism. *J Lipid Res*. 2020;61(4):470-9. doi:10.1194/jlr.R119000547.

69. Fehringer G, Brenner DR, Zhang ZF, Lee YA, Matsuo K, Ito H et al. Alcohol and lung cancer risk among never smokers: A pooled analysis from the international lung cancer consortium and the SYNERGY study. *Int J Cancer*. 2017;140(9):1976-84. doi:10.1002/ijc.30618.

70. Sobhani V, Taghizadeh M, Moshkani-Farahani M. Platelet indices and function response to two types of high intensity interval exercise and comparison with moderate intensity continuous exercise among men after coronary artery bypass graft: A randomized trial. *ARYA Atheroscler*. 2018;14(5):188-95. doi:10.22122/arya.v14i5.1780.

71. Friedenreich CM, Ryder-Burbidge C, McNeil J. Physical activity, obesity and sedentary behavior in cancer etiology: epidemiologic evidence and biologic mechanisms. *Mol Oncol*. 2021;15(3):790-800. doi:10.1002/1878-0261.12772.

72. Zou X, Wang R, Yang Z, Wang Q, Fu W, Huo Z et al. Family Socioeconomic Position and Lung Cancer Risk: A Meta-Analysis and a Mendelian Randomization Study. *Front Public Health*. 2022;10:780538. doi:10.3389/fpubh.2022.780538.

73. Zaccardi F, Rocca B, Pitocco D, Tanese L, Rizzi A, Ghirlanda G. Platelet mean volume, distribution width, and count in type 2 diabetes, impaired fasting glucose, and metabolic syndrome: a meta-analysis. *Diabetes Metab Res Rev*. 2015;31(4):402-10. doi:10.1002/dmrr.2625.

74. Yi ZH, Luther Y, Xiong GH, Ni YL, Yun F, Chen J et al. Association between diabetes mellitus and lung cancer: Meta-analysis. *Eur J Clin Invest*. 2020;50(10):e13332. doi:10.1111/eci.13332.

75. Xu Y, Guo Y. Platelet indices and blood pressure: a multivariable mendelian randomization study. *Thromb J*. 2023;21(1):31. doi:10.1186/s12959-023-00475-6.

76. Christakoudi S, Kakourou A, Markozannes G, Tzoulaki I, Weiderpass E, Brennan P et al. Blood pressure and risk of cancer in the European Prospective Investigation into Cancer and Nutrition. *Int J Cancer*. 2020;146(10):2680-93. doi:10.1002/ijc.32576.

77. Fatima K, Moeed A, Waqar E, Atif AR, Kamran A, Rizvi H et al. Efficacy of statins in treatment and development of non-alcoholic fatty liver disease and steatohepatitis: A systematic review and meta-analysis. *Clin Res Hepatol Gastroenterol*. 2022;46(4):101816. doi:10.1016/j.clinre.2021.101816.

78. McGill MR, Hinson JA. The development and hepatotoxicity of acetaminophen: reviewing over a century of progress. *Drug Metab Rev*. 2020;52(4):472-500. doi:10.1080/03602532.2020.1832112.

79. Friis S, Nielsen GL, Mellemkjaer L, McLaughlin JK, Thulstrup AM, Blot WJ et al. Cancer risk in persons receiving prescriptions for paracetamol: a Danish cohort study. *Int J Cancer*. 2002;97(1):96-101. doi:10.1002/ijc.1581.

80. Sennels HP, Jørgensen HL, Hansen AL, Goetze JP, Fahrenkrug J. Diurnal variation of hematology parameters in healthy young males: the Bispebjerg study of diurnal variations. *Scand J Clin Lab Invest*. 2011;71(7):532-41. doi:10.3109/00365513.2011.602422.

81. Arredondo ME, Aranda E, Astorga R, Brennan-Bourdon LM, Campelo MD, Flores S et al. Breakfast can Affect Routine Hematology and Coagulation Laboratory Testing: An Evaluation on Behalf of COLABIOCLI WG-PRE-LATAM. *TH Open*. 2019;3(4):e367-e76. doi:10.1055/s-0039-3401002.

82. Wen H, Lin X, Sun D. The association between different hormone replacement therapy use and the incidence of lung cancer: a systematic review and meta-analysis. *J Thorac Dis*. 2022;14(2):381-95. doi:10.21037/jtd-22-48.

83. Eknoyan G. Adolphe Quetelet (1796-1874)--the average man and indices of obesity. *Nephrol Dial Transplant*. 2008;23(1):47-51. doi:10.1093/ndt/gfm517.

84. Krakauer NY, Krakauer JC. An Anthropometric Risk Index Based on Combining Height, Weight, Waist, and Hip Measurements. *J Obes*. 2016;2016:8094275. doi:10.1155/2016/8094275.
